# Supplementary material for: Association of Polyphenols Consumption with Risk for Gestational Diabetes Mellitus and Preeclampsia: A Systematic Review and Meta-Analysis
Source: Antioxidants (Basel). 2022 Nov 19;11(11):2294. doi: 10.3390/antiox11112294 (PMC9687635; doi:10.3390/antiox11112294)
Supplement: Supplementary file 1 [file antioxidants-11-02294-s001.zip › antioxidants-2022307-supplementary.pdf]

Supplementary Table S1. PRISMA checklist.

Section/topic	#	Checklist item	Reported on page #
**TITLE**			
Title	1	Identify the report as a systematic review, meta-analysis, or both.	1
**ABSTRACT**			
Structured summary	2	Provide a structured summary including, as applicable: background; objectives; data sources; study eligibility criteria, participants, and interventions; study appraisal and synthesis methods; results; limitations; conclusions and implications of key findings; systematic review registration number.	2
**INTRODUCTION**			
Rationale	3	Describe the rationale for the review in the context of what is already known.	3
Objectives	4	Provide an explicit statement of questions being addressed with reference to participants, interventions, comparisons, outcomes, and study design (PICOS).	4
**METHODS**			
Protocol and registration	4-7	Indicate if a review protocol exists, if and where it can be accessed (e.g., Web address), and, if available, provide registration information including registration number.	Protocol detailed in the Methods section page 4-7<br>Not registered
Eligibility criteria	5	Specify study characteristics (e.g., PICOS, length of follow-up) and report characteristics (e.g., years considered, language, publication status) used as criteria for eligibility, giving rationale.	5
Information sources	4	Describe all information sources (e.g., databases with dates of coverage, contact with study authors to identify additional studies) in the search and date last searched.	4
Search		Present full electronic search strategy for at least one database, including any limits used, such that it could be repeated.	Suppl Table 3
Study selection	5	State the process for selecting studies (i.e., screening, eligibility, included in systematic review, and, if applicable, included in the meta-analysis).	5
Data collection process	6	Describe method of data extraction from reports (e.g., piloted forms, independently, in duplicate) and any processes for obtaining and confirming data from investigators.	6
Data items	7-11	List and define all variables for which data were sought (e.g., PICOS, funding sources) and any assumptions and simplifications made.	7-10
Risk of bias in individual studies	7-11	Describe methods used for assessing risk of bias of individual studies (including specification of whether this was done at the study or outcome level), and how this information is to be used in any data synthesis.	7-10
Summary measures	10-11	State the principal summary measures (e.g., risk ratio, difference in means).	10-11
Synthesis of results	14	Describe the methods of handling data and combining results of studies, if done, including measures of consistency (e.g., I^2) for each meta-analysis.	10-11

From: Moher D, Liberati A, Tetzlaff J, Altman DG, The PRISMA Group (2009). Preferred Reporting Items for Systematic Reviews and Meta-Analyses: The PRISMA Statement. PLoS Med 6(7): e1000097. doi:10.1371/journal.pmed1000097

Supplementary Table S2. The electronic search string in different databases.

Database	Search string			Field labels	Start date	Last date of search	Number of hits
**PubMed Medline**	1	Curcumin/	11750	• exp/ = exploded MeSH term<br>• / = non exploded MeSH term<br>• .ti,ab,kf. = title, abstract and author keywords<br>• adjx = within x words, regardless of order<br>• * = truncation of word for alternate endings	unbound	May 2022	Polyphenols Pregnancy complications: 3,085<br>Diet Pregnancy complications: 7,627
	2	Coumaric Acids/	4844				
	3	exp Flavonoids/	114145				
	4	Phenols/	52178				
	5	exp Polyphenols/	21677				
	6	exp Tannins/	10788				
	7	(anthocyani* or benzoflavone? or biflavonoid* or bioflavonoid* or catechin* or catechuic acid* or catergen* or chalcon* or cianidanol* or coumarate or coumaric acid* or curcumin or cyanidanol* or diferuloylmethane or dikvertin* or epicatechin* or flavanone? or flavonoid* or flavone? or flavonol* or homoisoflavon* or hydroxycinnamic acid* or isoflavone? or leucoanthocyanidin* or phenolic acid* or phenol? or phlobatannin* or polyphenol* or proanthocyanidin* or quercetin* or resveratrol* or SRT 501 or SRT501 or stilbenetriol or tannin or tannins or tannic acid* or trihydroxystilbene or turmeric yellow).ti,ab,kf.	201007				
	8	or/1-7	275996				
	9	Chocolate/ or Cacao/ or Coffee/	10847				
	10	Tea/	10837				
	11	Fruit/	46872				
	12	exp Vegetables/	32654				

	13	(almond? or apple? or artichoke* or bean or beans or berries or berry or blackberr* or black currant? or blueberr* or chicory or chocolate or coffee or cocoa powder or fruit* or grapes or hazelnut? or mulberr* or nut? or pecan? or plum? or red onion* or spinach or soy or soybean? or strawberr* or sweet cherr* or tea or vegetable* or walnut?).ti,ab,kf.	324387				
	14	or/9-13	342546				
	15	8 or 14	574447				
	16	exp Pregnancy Complications/	441441				
	17	((diabet* or glucose*) adj5 (gravidarum or gestational or maternal or	29964				
	18	((arterial pressure or hypertens*) adj2 (maternal or pregnan* or gestetional)).ti,ab,kf.	10496				
	19	(EPH or edema-proteinuria-hypertension gestosis or hypertension-edema-proteinuria gestosis or preeclampsia or pre-eclampsia or pregnancy complication* or (pregnan* adj2 toxemia*) or proteinuria-edema-hypertension gestosis).ti,ab,kf.	57495				
	20	Fetal Growth Retardation/	16910				
	21	Infant, Small for Gestational Age/	7803				
	22	((f?etal or fetus or in utero or intrauterine or prenatal) adj3 growth).ti,ab,kf.	33386				
	23	((small or large) adj3 (date or gestational age)).ti,ab,kf.	15033				
	24	or/16-23	482952				
	25	15 and 24	3058				
	26	Diet/	168111				

	27	Diet, Mediterranean/	3972				
	28	((intake or consum*) adj3 (diet* or food or nutrition*)).ti,ab,kf.	134967				
	29	(eat or eating or ingest*).ti,ab,kf.	200843				
	30	mediterranean diet*.ti,ab,kf.	5877				
	31	or/26-30	449882				
	32	24 and 31	7627				
**Embase**	#1	'curcumin'/exp	27,948	• /exp = exploded Emtree term<br>• /de = non exploded Emtree term<br>• /mj = major focus<br>• ti,ab,kw = title, abstract and author keywords<br>• NEAR/x = within x words, regardless of order<br>• * = truncation of word for alternate endings	unbound	May 2022	Polyphenols Pregnancy complications: 2,082<br>Diet Pregnancy complications: 4,134
	#2	'coumaric acid'/de	5,197				
	#3	'flavonoid'/exp	166,187				
	#4	'phenol derivative'/de	60,603				
	#5	polyphenol'/de	22,611				
	#6	'tannin derivative'/exp	24,460				
	#7	anthocyani*:ti,ab,kw OR benzoflavone$:ti,ab,kw OR biflavonoid*:ti,ab,kw OR bioflavonoid*:ti,ab,kw OR catechin*:ti,ab,kw OR 'catechuic acid*':ti,ab,kw OR catergen*:ti,ab,kw OR chalcon*:ti,ab,kw OR cianidanol*:ti,ab,kw OR 'coumaric acid*':ti,ab,kw OR curcumin$:ti,ab,kw OR cyanidanol*:ti,ab,kw OR diferuloylmethane:ti,ab,kw OR dikvertin*:ti,ab,kw OR epicatechin*:ti,ab,kw OR flavanone$:ti,ab,kw OR flavonoid*:ti,ab,kw OR flavone$:ti,ab,kw OR flavonol*:ti,ab,kw OR homoisoflavon*:ti,ab,kw OR 'hydroxycinnamic acid*':ti,ab,kw OR isoflavone$:ti,ab,kw OR leucoanthocyanidin*:ti,ab,kw OR 'phenolic acid*':ti,ab,kw OR phenol$:ti,ab,kw OR phlobatannin*:ti,ab,kw OR polyphenol*:ti,ab,kw OR proanthocyanidin*:ti,ab,kw OR quercetin*:ti,ab,kw OR resveratrol*:ti,ab,kw OR 'srt 501':ti,ab,kw OR srt501:ti,ab,kw OR stilbenetriol:ti,ab,kw OR tannin:ti,ab,kw OR tannins:ti,ab,kw OR 'tannic acid*':ti,ab,kw OR	259,024				

		trihydroxystilbene:ti,ab,kw OR 'turmeric yellow':ti,ab,kw						
	#8	#1 OR #2 OR #3 OR #4 OR #5 OR #6 OR #7	358,077					
	#9	'chocolate'/de	1,769					
	#10	coffee'/exp	16,077					
	#11	'fruit'/exp	150,974					
	#12	'tea'/de	20,917					
	#13	'vegetable'/exp	220,123					
	#14	almond$:ti,ab,kw OR apple$:ti,ab,kw OR artichoke*:ti,ab,kw OR bean:ti,ab,kw OR beans:ti,ab,kw OR berries:ti,ab,kw OR berry:ti,ab,kw OR blackberr*:ti,ab,kw OR ‘black currant$’:ti,ab,kw OR blueberr*:ti,ab,kw OR chicory:ti,ab,kw OR chocolate:ti,ab,kw OR coffee:ti,ab,kw OR 'cocoa powder':ti,ab,kw OR fruit*:ti,ab,kw OR grapes:ti,ab,kw OR hazelnut$:ti,ab,kw OR mulberr*:ti,ab,kw OR nut$:ti,ab,kw OR pecan$:ti,ab,kw OR plum$:ti,ab,kw OR 'red onion*':ti,ab,kw OR spinach:ti,ab,kw OR soy:ti,ab,kw OR soybean$:ti,ab,kw OR strawberr*:ti,ab,kw OR 'sweet cherr*':ti,ab,kw OR tea:ti,ab,kw OR vegetable*:ti,ab,kw OR walnut$:ti,ab,kw	379,983					
	#15	#9 OR #10 OR #11 OR #12 OR #13 OR #14	547,989					
	#16	#8 OR #15	833,708					
	#17	'pregnancy complication'/exp	158,645					
	#18	((diabet* OR glucose*) NEAR/3 (gravidarum OR gestational OR maternal OR pregnan*)):ti,ab,kw	40,990					
	#19	(('arterial pressure' OR hypertens*) NEAR/2 (matern* OR pregnan* OR gestetional)):ti,ab,kw	15,360					
	#20	eph:ti,ab,kw OR 'edema-proteinuria-hypertension gestosis':ti,ab,kw OR	61,154					

		'hypertension-edema-proteinuria gestosis':ti,ab,kw OR preeclamp*:ti,ab,kw OR 'pre-eclamp*':ti,ab,kw OR 'pregnancy complication':ti,ab,kw OR ((pregnan* NEAR/2 toxemia*):ti,ab,kw) OR 'proteinuria-edema-hypertension gestosis':ti,ab,kw					
	#21	'intrauterine growth retardation'/de	30,572				
	#22	'large for gestational age'/de	3,645				
	#23	'small for date infant'/de	16,830				
	#24	((f$etal OR fetus OR 'in utero' OR intrauterine OR prenatal) NEAR/3 growth):ti,ab,kw	47,260				
	#25	((small OR large) NEAR/3 (date OR 'gestational age')):ti,ab,kw	20,880				
	#26	#17 OR #18 OR #19 OR #20 OR #21 OR #22 OR #23 OR #24 OR #25	270,328				
	#27	#16 AND #26	2082				
	#28	'diet'/mj	81,698				
	#29	'mediterranean diet'/de	9,254				
	#30	((intake OR consum*) NEAR/3 (diet* OR food OR nutrition*)):ti,ab,kw	176,220				
	#31	eat:ti,ab,kw OR eating:ti,ab,kw OR ingest*:ti,ab,kw	262,758				
	#32	mediterranean diet*':ti,ab,kw	8,357				
	#33	#28 OR #29 OR #30 OR #31 OR #32	484,028				
	#34	#26 AND #33	4134				
**Web of Science**	#1	TS=(anthocyani* OR benzoflavone? OR biflavonoid* OR bioflavonoid* OR catechin* OR catechuic acid* OR catergen* OR chalcon* OR cianidanol* OR "coumarate" OR "coumaric acid*" OR "curcumin" OR cyanidanol* OR diferuloylmethane OR dikvertin* OR epicatechin* OR flavanone? OR flavonoid* OR flavone? OR flavonol* OR homoisoflavon* OR "hydroxycinnamic acid*" OR isoflavone? OR	345,089	• TS/Topic = title, abstract, author keywords and Keywords Plus<br>• NEAR/x = within x words, regardless of order<br>• * = truncation of word for	unbound	May 2022	Polyphenols Pregnancy complications: 996<br><br>Diet Pregnancy complications: 1,956

		leucoanthocyanidin* OR "phenolic acid*" OR phenol? OR phlobatannin* OR polyphenol* OR proanthocyanidin* OR quercetin* OR resveratrol* OR "SRT 501" OR "SRT501" OR "stilbenetriol" OR "tannin" OR "tannins" OR "tannic acid*" OR trihydroxystilbene OR "turmeric yellow")		alternate endings			
	#2	TS=(almond? OR apple? OR artichoke* OR "bean" OR "beans" OR "berries" OR "berry" OR blackberr* OR "black currant?" OR blueberr* OR "chicory" OR "chocolate" OR "coffee" OR "cocoa powder" OR fruit* OR "grapes" OR hazelnut? OR mulberr* OR nut? OR pecan? OR plum? OR "red onion*" OR "spinach" OR "soy" OR soybean? OR strawberr* OR" sweet cherr*" OR "tea" OR vegetable* OR walnut?)	763,799				
	#3	#2 OR #1	1,027,554				
	#4	TS=((diabet* OR glucose*) NEAR/4 ("gravidarum" OR "gestational" OR "maternal" OR pregnan*))	32,445				
	#5	TS=(("arterial pressure" OR hypertens*) NEAR/1 ("maternal" OR pregnan* OR "gestetional"))	10,969				
	#6	TS=((EPH OR "edema-proteinuria-hypertension gestosis" OR "hypertension-edema-proteinuria gestosis" OR "preeclampsia" OR "pre-eclampsia" OR "pregnancy complication*" OR (pregnan* NEAR/1 toxemia*) OR "proteinuria-edema-hypertension gestosis"))	53,486				
	#7	TS=((f?etal OR "fetus" OR "in utero" OR "intrauterine" OR "prenatal") NEAR/2 "growth")	22,090				
	#8	TS=(("small" OR "large") NEAR/2 ("date" OR "gestational age"))	14,912				

	#9	#8 OR #7 OR #6 OR #5 OR #4	112,832				
	#10	#9 AND #3	996				
	#11	TS=(("intake" OR consum*) NEAR/2 (diet* OR "food" OR nutrition*))	176,025				
	#12	TS=("eat" OR "eating" OR ingest*)	248,083				
	#13	TS="mediterranean diet*"	10,578				
	#14	#13 OR #12 OR #11	405,354				
	#15	#14 AND #9	1956				

Supplementary Table S3. The Newcastle-Ottawa Scale (NOS) for assessing the quality of non-randomized studies in meta-analysis.

PE Studies	Year published	Selection	Comparability	Outcome	Score
Triche et al,	2008	****	**	***	**9**
Wei et al,	2009	***	*	***	**7**
Saftlas et al,	2010	****	*	-	**5**

Kanawishi et al,	2021	***	*	**	**6**
**PE Studies**	**Year published**	**Selection**	**Comparability**	**Outcome**	**Score**
Adeney et al,	2007	*****	*	***	**9**
Hinkle et al,	2015	****	**	***	**9**
Tryggvadottir et al,	2016	***	**	**	**7**
Huang et al,	2017	***	**	**	**7**
Dong et al,	2019	****	**	***	**9**
Dong et al,	2021	***	*	**	**6**
Gao et al,	2021	****	*	**	**7**
Sun et al,	2021	****	*	**	**7**

Scale: a maximum of 4 stars can be awarded for selection, 2 for comparability and 3 for outcomes. A score of ≥6 indicates a high-quality study. *: each asterisk (*) represents a point score for the scale.

Supplementary Table S4. MOOSE checklist

**Reporting Criteria**	**Reported (Yes/No)**	**Reported on Page No.**
**Reporting of Background**		
Problem definition	Yes	4
Hypothesis statement	No	NA
Description of Study Outcome(s)	Yes	4
Type of exposure or intervention used	Yes	4

Type of study design used	Yes	4-6
Study population	Yes	4
**Reporting of Search Strategy**		
Qualifications of searchers (e.g., librarians and investigators)	Yes	5
Search strategy, including time period included in the synthesis and keywords	Yes	5
Effort to include all available studies, including contact with authors	No	NA
Databases and registries searched	Yes	5
Search software used, name and version, including special features used (e.g., explosion)	No	NA
Use of hand searching (e.g., reference lists of obtained articles)	Yes	5
List of citations located and those excluded, including justification	Yes	Table 1, 2 and Suppl. Table 3
Method for addressing articles published in languages other than English	No	NA
Method of handling abstracts and unpublished studies	No	NA
Description of any contact with authors	No	NA
**Reporting of Methods**		
Description of relevance or appropriateness of studies assembled for assessing the hypothesis to be tested	Yes	5
Rationale for the selection and coding of data (e.g., sound clinical principles or convenience)	Yes	5
Documentation of how data were classified and coded (e.g., multiple raters, blinding, and interrater reliability)	Yes	6
Assessment of confounding (e.g., comparability of cases and controls in studies where appropriate)	No	NA
Assessment of study quality, including blinding of quality assessors; stratification or regression on possible predictors of study results	Yes	6
Assessment of heterogeneity	Yes	7
Description of statistical methods (e.g., complete description of fixed or random effects models, justification of whether the chosen models account for predictors of study results, dose-response models, or cumulative meta-analysis) in sufficient detail to be replicated	Yes	7
Provision of appropriate tables and graphics	Yes	Fig. 1
**Reporting of Results**		
Table giving descriptive information for each study included	Yes	18-21 (Table 1)
Results of sensitivity testing (e.g., subgroup analysis)	Yes	11
Indication of statistical uncertainty of findings	Yes	11
**Reporting of Discussion**		
Quantitative assessment of bias (e.g., publication bias)	No	NA
Justification for exclusion (e.g., exclusion of non–English-language citations)	Yes	15, Supp Table 3
Assessment of quality of included studies	Yes	Supp Table 2
**Reporting of Conclusions**		
Consideration of alternative explanations for observed results	No	NA

Generalization of the conclusions (i.e., appropriate for the data presented and within the domain of the literature review)	Yes	16
Guidelines for future research	Yes	16-17
Disclosure of funding source	Yes	17

*Stroup DF, Berlin JA, Morton SC, et al, for the Meta-analysis Of Observational Studies in Epidemiology (MOOSE) Group. Meta-analysis of Observational Studies in Epidemiology. A Proposal for Reporting. JAMA. 2000;283(15):2008-2012. doi: 10.1001/jama.283.15.2008.

Supplementary Table S5. Excluded, full text assessed studies with a reason for exclusion from the systematic review and meta-analysis.

N	Studies	Reason for exclusion
1	Abbasi R, Bakhshimoghaddam F, Alizadeh M. J. Maternal Fetal Major dietary patterns in relation to preeclampsia among Iranian pregnant women: a case-control study. Neonatal Med. 2021 Nov;34(21):3529-3536.	Specific food source of polyphenols were not analyzed
2	Amiruddin R, Asrianti T, Abdullah MT. Fiber, Coffee, Cigarette and Gestational Diabetes Mellitus in Makassar Indonesia. Asian Journal of Epidemiology. 2017; 10(1): 26-31.	Study design: cross-sectional

3	Bakker R, Steegers EA, Raat H, Hofman A, Jaddoe VW. Maternal caffeine intake, blood pressure, and the risk of hypertensive complications during pregnancy. The Generation R Study. Am J Hypertens. 2011 Apr;24(4):421-8. doi: 10.1038/ajh.2010.242. Epub 2010 Dec 16.	Analysis was by quantity of caffeine and not of quantity of polyphenols
4	Balbi MA, Crivellenti LC, Zuccolotto DCC, Franco LJ, Sartorelli DS. The relationship of flavonoid intake during pregnancy with excess body weight and gestational diabetes mellitus. Arch Endocrinol Metab. 2019 May 30;63(3):241-249.	Study design: cross-sectional
5	Bao W, Bowers K, Tobias DK, Hu FB, Zhang C. Pre-pregnancy dietary protein intake, major dietary protein sources, and the risk of gestational diabetes mellitus: a prospective cohort study. Diabetes Care. 2013 Jul;36(7):2001-8. doi: 10.2337/dc12-2018. Epub 2013 Feb 1.	Analysis of protein intake and not polyphenols
6	Bao W, Tobias DK, Hu FB, Chavarro JE, Zhang C. Pre-pregnancy potato consumption and risk of gestational diabetes mellitus: prospective cohort study. BMJ. 2016 Jan 12;352:h6898. doi: 10.1136/bmj.h6898.	Analysis of potato consumption and not polyphenols
7	Basu A, Crew J, Ebersole JL, Kinney JW, Salazar AM, Planinic P, Alexander JM. Dietary Blueberry and Soluble Fiber Improve Serum Antioxidant and Adipokine Biomarkers and Lipid Peroxidation in Pregnant Women with Obesity and at Risk for Gestational Diabetes. Antioxidants (Basel). 2021 Aug 22;10(8):1318.	Effect of berry consumption on GDM diagnosis was not analyzed
8	Bech BH, Frydenberg M, Henriksen TB, Obel C, Olsen J. Coffee Consumption During Pregnancy and Birth Weight: Does Smoking Modify the Association? Journal of caffeine research 2015; 5 (2).	Nor PE or GDM risk analyzed
9	Bujold E, Leblanc V, Lavoie-Lebel É, Babar A, Girard M, Poungui L, Blanchet C, Marc I, Lemieux S, Belkacem A, Sidi EL, Dodin S. High-flavanol and high-theobromine versus low-flavanol and low-theobromine chocolate to improve uterine artery pulsatility index: a double blind randomized clinical trial. J Matern Fetal Neonatal Med. 2017 Sep;30(17):2062-2067.	Nor PE or GDM risk analyzed
10	Chen L, Hu FB, Yeung E, Tobias DK, Willett WC, Zhang C. Prepregnancy consumption of fruits and fruit juices and the risk of gestational diabetes mellitus: a prospective cohort study. Diabetes Care. 2012 May;35(5):1079-82. doi: 10.2337/dc11-2105.	Analysis of fruits and fruit juice consumption not clear, do not indicate the follow up.
11	Daneshzad E, Tehrani H, Bellissimo N, Azadbakht L. Dietary Total Antioxidant Capacity and Gestational Diabetes Mellitus: A Case-Control Study. Oxid Med Cell Longev. 2020 Oct 8;2020:5471316. doi: 10.1155/2020/5471316.	Analysis did not consider the consumption of polyphenols.
12	de la Torre NG, Assaf-Balut C, Jiménez Varas I, Del Valle L, Durán A, Fuentes M, Del Prado N, Bordiú E, Valerio JJ, Herraiz MA, Izquierdo N, Torrejón MJ, Cuadrado MA, de Miguel P,	Study about the Mediterranean diet and not of polyphenols.

	Familiar C, Runkle I, Barabash A, Rubio MA, Calle-Pascual AL. Effectiveness of Following Mediterranean Diet Recommendations in the Real World in the Incidence of Gestational Diabetes Mellitus (GDM) and Adverse Maternal-Fetal Outcomes: A Prospective, Universal, Interventional Study with a Single Group. The St Carlos Study. Nutrients. 2019 May 28;11(6):1210. doi: 10.3390/nu11061210.	
13	Endeshaw M, Abebe F, Bedimo M, Asart A. Diet and Pre-eclampsia: A Prospective Multicentre Case-Control Study in Ethiopia. Midwifery. 2015 Jun;31(6):617-24.	Estimator is confusing, do not measure polyphenol rich-foods.
14	Fang JH, Zhang SH, Yu XM & Yang Y. Effects of Quercetin and Melatonin in Pregnant and Gestational Diabetic Women. Lat. Am. J. Pharm. 35 (6): 1420-5 (2016)	Full publication is not available
15	Heazell AEP, Timms K, Scott RE, Rockliffe L, Budd J, Li M, Cronin R, McCowan LME, Mitchell EA, Stacey T, Roberts D, Thompson JMD. Associations between consumption of coffee and caffeinated soft drinks and late stillbirth-Findings from the Midland and North of England stillbirth case-control study. Eur J Obstet Gynecol Reprod Biol. 2021 Jan;256:471-477.	Nor PE or GDM risk analyzed
16	Izadi V, Tehrani H, Haghighatdoost F, Dehghan A, Surkan PJ, Azadbakht L. Adherence to the DASH and Mediterranean diets is associated with decreased risk for gestational diabetes mellitus. Nutrition. 2016 Oct;32(10):1092-6. doi: 10.1016/j.nut.2016.03.006. Epub 2016 Mar 19.	Study about Mediterranean diet and dietary approaches to stop hypertension (DASH) and not polyphenols.
17	Jarman M, Mathe N, Ramazani F, Pakseresht M, Robson PJ, Johnson ST, Bell RC; APrON and ENRICH study teams. Dietary Patterns Prior to Pregnancy and Associations with Pregnancy Complications. Nutrients. 2018 Jul 17;10(7):914. doi: 10.3390/nu10070914.	Analysis did not consider the consumption of polyphenols.
18	Karamanos B, Thanopoulou A, Anastasiou E, Assaad-Khalil S, Albache N, Bachaoui M, Slama CB, El Ghomari H, Jotic A, Lalic N, Lapolla A, Saab C, Marre M, Vassallo J, Savona-Ventura C; MGSD-GDM Study Group. Relation of the Mediterranean diet with the incidence of gestational diabetes. Eur J Clin Nutr. 2014 Jan;68(1):8-13. doi: 10.1038/ejcn.2013.177. Epub 2013 Oct 2.	Analysis considered consumption of Mediterranean diet and not consumption of polyphenols.
19	Kinnunen TI, Puhkala J, Raitanen J, Ahonen S, Aittasalo M, Virtanen SM, Luoto R. Effects of dietary counselling on food habits and dietary intake of Finnish pregnant women at increased risk for gestational diabetes - a secondary analysis of a cluster-randomized controlled trial. Matern Child Nutr. 2014 Apr;10(2):184-97. doi: 10.1111/j.1740-8709.2012.00426.x. Epub 2012 Jun 27.	Study considered the type of diet but not polyphenol consumption.
20	Mannucci C, Attard E, Calapai F, Facchinetti F, D'Anna R, Vannacci A, Santamaria A, Lenti MC, Righi M, Perone M,	Nor PE or GDM risk analyzed

	Sorbara EE, Alibrandi A, Oteri A, Inferrera G, Calapai G. Coffee intake during pregnancy and neonatal low birth weight: data from a multicenter Italian cross sectional study. J Matern Fetal Neonatal Med. 2020 Nov 18;1-5.	
21	Okubo H, Miyake Y, Tanaka K, Sasaki S, Hirota Y. Maternal total caffeine intake, mainly from Japanese and Chinese tea, during pregnancy was associated with risk of preterm birth: the Osaka Maternal and Child Health Study. Nutr Res. 2015 Apr;35(4):309-16.	Nor PE or GDM risk analyzed
22	Radesky JS, Oken E, Rifas-Shiman SL, Kleinman KP, Rich-Edwards JW, Gillman MW. Diet during early pregnancy and development of gestational diabetes. Paediatr Perinat Epidemiol. 2008 Jan;22(1):47-59. doi: 10.1111/j.1365-3016.2007.00899.x.	Study considered the type of diet and did not consider consumption of polyphenols.
23	Ruiz-Gracia T, Duran A, Fuentes M, Rubio MA, Runkle I, Carrera EF, Torrejón MJ, Bordiú E, Valle LD, García de la Torre N, Bedia AR, Montañez C, Familiar C, Calle-Pascual AL. Lifestyle patterns in early pregnancy linked to gestational diabetes mellitus diagnoses when using IADPSG criteria. The St Carlos gestational study. Clin Nutr. 2016 Jun;35(3):699-705. doi: 10.1016/j.clnu.2015.04.017. Epub 2015 May 8.	Study considered the type of food consumed and lifestyle. It did not consider consumption of polyphenols.
24	Schoenaker DA, Soedamah-Muthu SS, Callaway LK, Mishra GD. Pre-pregnancy dietary patterns and risk of gestational diabetes mellitus: results from an Australian population-based prospective cohort study. Diabetologia. 2015 Dec;58(12):2726-35. doi: 10.1007/s00125-015-3742-1. Epub 2015 Sep 10.	Analysis considered dietary patterns and not polyphenols.
25	Sharbaf, F. R., Dehghanpour, P., Shariat, M., & Dalili, H. (2013). Caffeine Consumption and Incidence of Hypertension in Pregnancy. Journal of Family & Reproductive Health, 7(3).	Analysis was by quantity of caffeine and not by quantity of polyphenols.
26	Tobias DK, Zhang C, Chavarro J, Bowers K, Rich-Edwards J, Rosner B, Mozaffarian D, Hu FB. Prepregnancy adherence to dietary patterns and lower risk of gestational diabetes mellitus. Am J Clin Nutr. 2012 Aug;96(2):289-95. doi: 10.3945/ajcn.111.028266. Epub 2012 Jul 3.	Analysis considered dietary patterns and not polyphenols.
27	Yusuf H, Subih HS, Obeidat BS, Sharkas G. Associations of macro and micronutrients and antioxidants intakes with preeclampsia: A case-control study in Jordanian pregnant women. Nutr Metab Cardiovasc Dis. 2019 May;29(5):458-466. doi: 10.1016/j.numecd.2019.01.008. Epub 2019 Jan 29.	Study considered dietary patterns and types of macro and micronutrients but did not consider consumption of polyphenols.

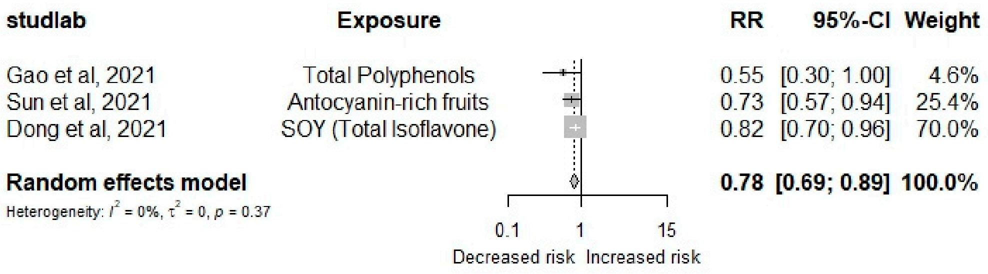
studlab
Exposure
RR
95%-CI
Weight
Gao et al, 2021
Total Polyphenols
0.55 [0.30; 1.00] 4.6%
Sun et al, 2021
Antocyanin-rich fruits
0.73 [0.57; 0.94] 25.4%
Dong et al, 2021
SOY (Total Isoflavone)
0.82 [0.70; 0.96] 70.0%
Random effects model
0.78 [0.69; 0.89] 100.0%
Heterogeneity: I2 = 0%, τ2 = 0, p = 0.37
0.1
1
15
Decreased risk
Increased risk

(A)

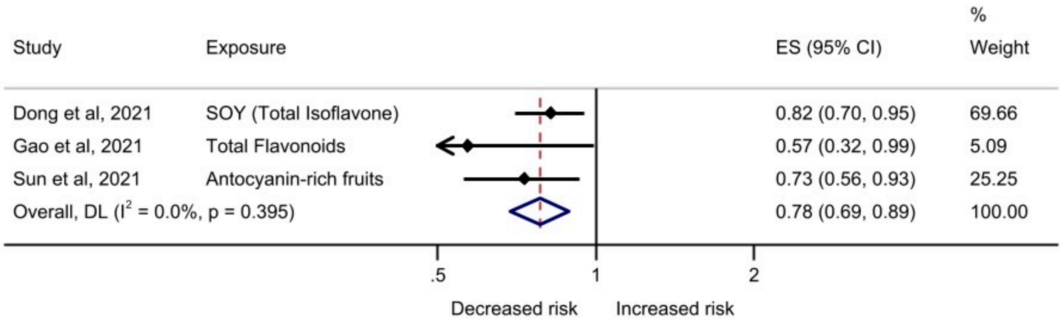
Study
Exposure
ES (95% CI)
% Weight
Dong et al, 2021
SOY (Total Isoflavone)
0.82 (0.70, 0.95)
69.66
Gao et al, 2021
Total Flavonoids
0.57 (0.32, 0.99)
5.09
Sun et al, 2021
Antocyanin-rich fruits
0.73 (0.56, 0.93)
25.25
Overall, DL (I2 = 0.0%, p = 0.395)
0.78 (0.69, 0.89)
100.00
.5
1
2
Decreased risk
Increased risk

(B)

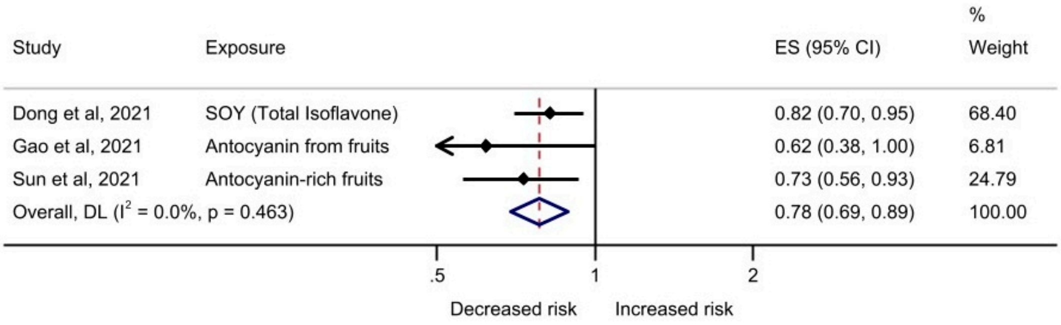
Study
Exposure
ES (95% CI)
% Weight
Dong et al, 2021
SOY (Total Isoflavone)
0.82 (0.70, 0.95)
68.40
Gao et al, 2021
Antocyanin from fruits
0.62 (0.38, 1.00)
6.81
Sun et al, 2021
Antocyanin-rich fruits
0.73 (0.56, 0.93)
24.79
Overall, DL (I2 = 0.0%, p = 0.463)
0.78 (0.69, 0.89)
100.00
.5
1
2
Decreased risk
Increased risk

(C)

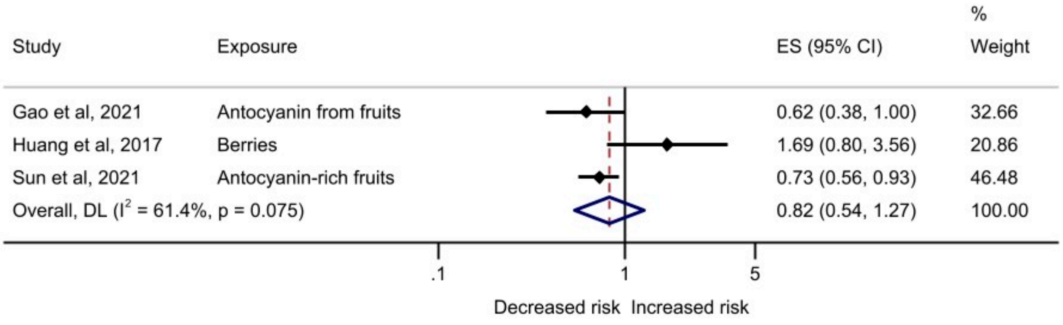
Study
Exposure
ES (95% CI)
% Weight
Gao et al, 2021
Antocyanin from fruits
0.62 (0.38, 1.00)
32.66
Huang et al, 2017
Berries
1.69 (0.80, 3.56)
20.86
Sun et al, 2021
Antocyanin-rich fruits
0.73 (0.56, 0.93)
46.48
Overall, DL (I2 = 61.4%, p = 0.075)
0.82 (0.54, 1.27)
100.00
.1
1
5
Decreased risk
Increased risk

(D)

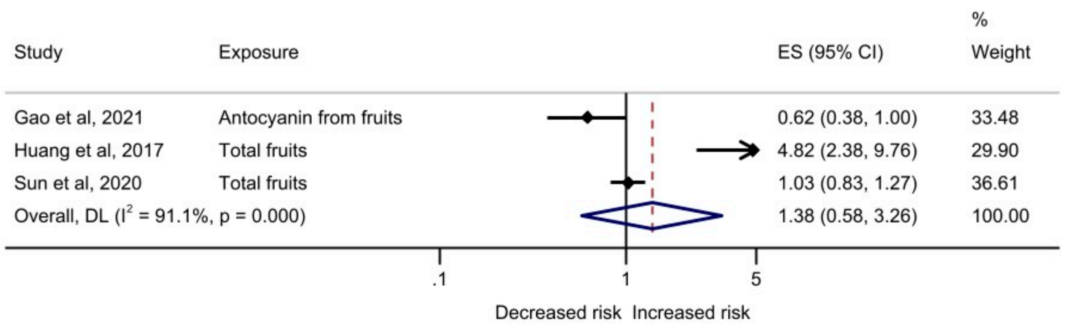

(E)

Supplementary Figure S1. Total polyphenol intake or their subclasses and GDM association. **Figure S1A.** Total polyphenol intake considering isoflavone, total polyphenols and anthocyanin-rich fruits and GDM association. **Figure S1B.** Total polyphenol intake considering isoflavone, total flavonoids and anthocyanin-rich fruits and GDM association. **Figure S1C.** Total polyphenol intake considering isoflavone, anthocyanins from fruits and anthocyanin-rich fruits and GDM association. **Figure S1D.** Total polyphenol intake considering berries, anthocyanins from fruits and anthocyanin-rich fruits and GDM association. **Figure S1E.** Total polyphenol intake considering anthocyanins from fruits and total fruits and GDM association.

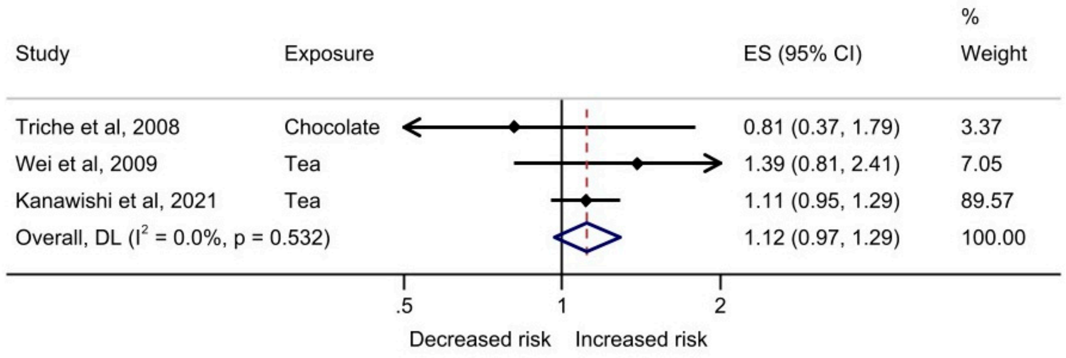

Supplementary Figure S2. Total polyphenol intake and PE association without gestational hypertension.
